# Concordance of Diagnosis of Autism Spectrum Disorder Made by Pediatricians vs a Multidisciplinary Specialist Team

**DOI:** 10.1001/jamanetworkopen.2022.52879

**Published:** 2023-01-25

**Authors:** Melanie Penner, Lili Senman, Lana Andoni, Annie Dupuis, Evdokia Anagnostou, Shawn Kao, Abbie Solish, Michelle Shouldice, Genevieve Ferguson, Jessica Brian

**Affiliations:** 1 Autism Research Centre, Bloorview Research Institute, Holland Bloorview Kids Rehabilitation Hospital, Toronto, Ontario, Canada; 2 Department of Paediatrics, University of Toronto, Toronto, Ontario, Canada; 3 Department of Psychology, University of Massachusetts, Boston; 4 Dalla Lana School of Public Health, University of Toronto, Toronto, Ontario, Canada; 5 Pediatricians Alliance of Ontario, Toronto, Ontario, Canada; 6 Paediatric Medicine, Hospital for Sick Children, Toronto, Ontario, Canada

## Abstract

**Importance:**

Wait times for autism spectrum disorder (ASD) diagnosis are lengthy because of inadequate supply of specialist teams. General pediatricians may be able to diagnose some cases of ASD, thereby reducing wait times.

**Objective:**

To determine the accuracy of ASD diagnostic assessments conducted by general pediatricians compared with a multidisciplinary team (MDT).

**Design, Setting, and Participants:**

This prospective diagnostic study was conducted in and a specialist assessment center in Toronto, Ontario, Canada, and Ontario general pediatrician practices from June 2016 to March 2020. Children were younger than 5.5 years, referred with a developmental concern, and without an existing ASD diagnosis. Data analysis was performed from October 2021 to February 2022.

**Exposures:**

The pediatrician and MDT each conducted blinded assessments and recorded a decision as to whether the child had ASD.

**Main Outcomes and Measures:**

Main outcomes included sensitivity, specificity, positive predictive value (PPV), and negative predictive value (NPV). A logistic regression was performed to identify factors associated with accurate pediatrician assessment for children with or without an ASD diagnosis.

**Results:**

Seventeen pediatricians (12 women [71%]) participated in the study and referred 106 children (79 boys [75%]; mean [SD] age, 41.9 [13.3] months). Sixty participants (57%) were from minoritized racial and ethnic groups (eg, Black, Asian, Hispanic, Middle Eastern, and multiracial). Seventy-two participants (68%) received a diagnosis of ASD by the MDT. Sensitivity and specificity of the pediatrician assessments compared with MDT were 0.75 (95% CI, 0.67-0.83) and 0.79 (95% CI, 0.62-0.91), respectively. The PPV of the pediatrician assessments was 0.89 (95% CI, 0.80-0.94) (ie, 89% agreement with the MDT), and NPV was 0.60 (95% CI, 0.49-0.70) (ie, 60% agreement with the MDT). Higher pediatrician certainty (odds ratio [OR], 3.33; 95% CI, 1.71-7.34; *P* = .001) was associated with increased diagnostic accuracy for children with ASD. Lower accuracy was seen for children with higher Visual Reception subscale developmental skills (OR, 0.93; 95% CI, 0.89-0.97; *P* = .001), speaking abilities (OR, 0.17; 95% CI, 0.03-0.67; *P* = .03), and White race (OR, 0.32; 95% CI, 0.10-0.97; *P* = .04). Age, gender, and Autism Diagnostic Observation Schedule, 2nd Edition composite scores were not significantly associated with the accuracy of assessments. All 7 children with a sibling with ASD received an accurate diagnosis; otherwise, no significant factors were identified for accuracy in children without ASD.

**Conclusions and Relevance:**

This study of concordance of autism assessment between pediatricians and an expert MDT in young children found high accuracy when general pediatricians felt confident and lower accuracy when ruling out ASD. These findings suggest that children with co-occurring delays may be potential candidates for community assessment.

## Key Points

**Question:**

How accurate are general pediatricians at assessing children for autism spectrum disorder (ASD) compared with a multidisciplinary team?

**Findings:**

This diagnostic study enrolled 17 general pediatricians and 106 children with possible ASD who were referred to them. When the pediatrician thought ASD was present, the team agreed 89% of the time; when the pediatrician did not think ASD was present, the team agreed only 60% of the time.

**Meaning:**

The concordance between pediatrician diagnosis of ASD and that of an expert team was high, but when ruling out ASD, the concordance was lower; additionally, children with co-occurring delays are potential candidates for community assessment.

## Teaser

This diagnostic study examines the accuracy of autism spectrum disorder (ASD) diagnostic assessments conducted by general pediatricians compared with a multidisciplinary team.

## Introduction

Timely autism spectrum disorder (ASD) diagnosis facilitates access to supports at young ages, which positively impacts development,^1^ reduces family distress,^2,3^ and improves outcomes.^4^ Many guidelines^5,6,7^ recommend multidisciplinary teams (MDTs) for all ASD diagnostic assessments to inform the ASD diagnosis and subsequent therapeutic strategies. This model is resource intensive and cannot meet the demand for assessments, contributing to wait times^8^ and delaying eligibility for ASD therapies. Expanding capacity at the general pediatrician level may improve access to ASD diagnosis and facilitate entry to therapy when it is most effective^1,9^; however, this strategy depends on the ability of general pediatricians to accurately diagnose autism.

ASD is a neurodevelopmental disorder that is characterized by challenges in social communication and interaction and the presence of restrictive, repetitive behaviors, interests, or activities.^10^ There is no single, objective, criterion standard test for ASD, and the diagnosis is based on clinicians’ best estimates, often informed by standardized tests. ASD diagnostic guidelines have traditionally recommended MDT assessments^5,6,7^; however, in the absence of empirical evidence, these recommendations were developed largely on the basis of expert opinion.^11^ Recent guidelines have recommended that children with clear-cut or uncomplicated presentations of ASD can undergo streamlined assessment.^12,13^ The increasing prevalence of ASD (1 of 44 children in the US^14^ and 1 of 66 children in Canada^15^) has contributed to lengthy wait times for diagnosis, delaying access to supports. The median clinician-reported wait time from referral to receipt of ASD diagnosis in Canada was previously reported as 7 months^14^; this has likely lengthened since the COVID-19 pandemic.

Of late, there has been interest in whether general pediatricians can diagnose some cases of ASD.^16^ Expanding the diagnostic workforce can facilitate timelier access to diagnosis and intervention; however, these assessments must be accurate. There have been few studies evaluating the accuracy of ASD diagnosis by different types of clinicians. One retrospective study^17^ from the era of *Diagnostic and Statistical Manual of Mental Disorders* (Fourth Edition, Text Revision) diagnostic subtypes found an odds ratio (OR) of 1.73 (95% CI, 1.31-2.29) for children whose ASD was diagnosed by a pediatrician compared with those whose ASD was diagnosed by a MDT,^18^ which is difficult to interpret in the *Diagnostic and Statistical Manual of Mental Disorders* (Fifth Edition) ASD conceptualization. One Utah-based study^15^ evaluated general pediatrician diagnostic accuracy for a small subset of 18 patients as part of a clinical program and found greater than 90% agreement and a 44% reduction in wait times. Other studies^17,19^ have evaluated diagnostic accuracy after training programs and have found high rates of agreement. To our knowledge, there have been no prospective studies with a primary objective of determining the accuracy of general pediatricians in assessing for ASD. The objectives of the present study were to (1) determine the accuracy of general pediatrician ASD diagnostic assessment compared with subspecialist MDT assessment and (2) determine factors associated with accurate general pediatrician diagnostic assessment.

## Methods

### Design

This study used a prospective diagnostic design, measuring agreement between general pediatrician ASD assessment and a subspecialist MDT assessment. MDTs are considered the highest standard for ASD assessment,^5^ and we considered them the accurate comparator. Patient participants were randomly allocated to 2 groups: a group who had their MDT visits before their pediatrician assessment and a group who had their MDT visits after their pediatrician assessment. This was done to balance a learning effect of parents, who might report their child’s symptoms differently after undergoing the first assessment. This study was performed between June 2016 and March 2020, when the study was stopped because of the COVID-19 pandemic and subsequent limitations on in-person assessments. Ethics approval was obtained from Holland Bloorview Kids Rehabilitation Hospital. Parents and/or guardians of the children provided written informed consent. Study design and methods are informed by Standards for Reporting of Diagnostic Accuracy (STARD) reporting guidelines.^18^

### Setting and Participants

In Canada, all residents have access to government-funded health care, including physician assessments. Most children receive their primary care from family physicians or nurse practitioners and are referred to consultant general pediatricians if there is a concern for which the family physician requires consultation. This pediatrician frequently then refers the child for a subspecialist diagnostic assessment if there is possible ASD.^14,16^

There were 2 groups of participants: (1) general pediatricians and (2) patients referred to the general pediatrician by primary care physicians. All practicing general pediatricians were eligible for inclusion unless they had completed a subspecialty training program in developmental pediatrics or did not receive at least 1 referral per month for a child with possible ASD. General pediatrician participants recruited consecutive patients (maximum, 10 patients per pediatrician) younger than 5.5 years who were referred with any of the following: (1) failed 18-month developmental screen (other than isolated motor delay); (2) speech or language delay; (3) social or emotional delay or concern; (4) behavioral, inattention, hyperactivity, or impulsivity concerns; (5) global developmental delay or concern; or (6) referral with a query of ASD. Children with an existing ASD diagnosis were excluded.

### Procedure

General pediatricians conducted their ASD assessment as they typically would but without informing families of their diagnostic impression. MDT appointments occurred no more than 4 weeks before or after the pediatrician visit to ensure the assessments were contemporaneous. The MDT consisted of a developmental pediatrician (M.P.), psychologist (J.B. or A.S.), and a psychometrist (L.S. or L.A.). The developmental pediatrician was consistent through all cases. The MDT assessment included the Autism Diagnostic Observation Schedule, 2nd Edition (ADOS-2)^20^ for all children. Developmental level was assessed with the Mullen Scales of Early Learning (MSEL).^21^ Both the ADOS-2 and MSEL were conducted by a psychometrist who had achieved research level reliability. Of note, some children were not able to complete the full MSEL; for this reason, the Visual Reception subscale was prioritized for administration as an early proxy for nonverbal intelligence quotient.^22^ Parents of children with a developmental age of 18 months or greater completed the Autism Diagnostic Interview–Revised^23^ with a psychometrist who had achieved research reliability. If the child’s developmental age was below 18 months, the developmental pediatrician conducted a developmental and ASD-focused interview with the family. The developmental pediatrician also gathered information about family history and conducted a physical examination with the child. If additional information was needed, the developmental pediatrician collected information about the child from a daycare worker or teacher.

The MDT met after their assessment to discuss their clinical impressions (including test results) and documented their decision. The pediatrician sent their opinion to the MDT. The MDT unblinded themselves to the pediatrician’s opinion only after making their decision. Unblinding occurred to ensure the MDT was aware of possible sensitivities when communicating the diagnostic opinion to the pediatrician. When both assessments were complete, the MDT met with the family to discuss the results of the MDT assessment, including a diagnosis if applicable. The MDT also helped connect families to services. At the end of general pediatrician’s participation, a member of the MDT met with them to debrief on cases and provide a learning opportunity.

### Study Measures

Pediatrician participants made a forced decision on whether they believed the child met the diagnostic criteria for ASD, as well as a 5-point Likert scale indicating their level of certainty. They indicated their diagnosis and likely actions had the patient not been in the study: determine that the child does have ASD, determine the child does not have ASD, give a different diagnosis, refer to a subspecialist, or watch and wait. The MDT completed the same diagnostic decision and 5-point Likert certainty rating.

Pediatrician participants provided demographic information, including their age, gender, and years in practice, as well as information about their practice, including their wait times, the number of patients with autism they see, and the tools they use in ASD assessment. Demographic information for patient participants was reported by parents and included age, gender, race, ethnocultural background, and the presence of a sibling with autism. Race and ethnicity were evaluated because previous research has shown disparities in recognition of ASD in some minoritized groups compared to White children.^24^

### Statistical Analysis

Data analysis was performed from October 2021 to February 2022. Demographic information about both the pediatrician and patient samples were calculated using means with SDs for continuous data and proportions for categorical data. Descriptive statistics were reported for general pediatrician diagnosis (ASD vs not ASD) and likely actions outside the study context, as well as pediatrician and MDT levels of certainty. The primary outcomes for this study were accuracy data for general pediatrician diagnosis, including sensitivity, specificity, positive predictive value (PPV), and negative predictive value (NPV). We also report overall percentage agreement and the κ statistic for agreement.

We performed logistic regression analyses to identify factors associated with pediatrician agreement with an MDT diagnosis of ASD and with an MDT diagnosis of non-ASD. Tested factors included child age, gender, race and ethnocultural background (White vs minoritized racial and ethnic groups), whether the child was speaking or nonspeaking, having a sibling with autism, observed ASD features (ADOS-2 calibrated severity score), MSEL Visual Reception score, and pediatrician certainty. *P* < .05 was considered statistically significant. Data were analyzed using R statistical software version 4.1.1 (R Project for Statistical Computing).

## Results

### Child Sample

In total, 106 children and 17 pediatricians participated in the study. The majority of children (Table 1) were boys (79 children [75%]), with a mean (SD) age of 41.9 (13.3) months (range, 17-66 months). Race and ethnicity of the sample are reported in Table 1. Sixty participants (57%) were from minoritized racial and ethnic groups (eg, Black, Asian, Hispanic, Middle Eastern, and multiracial). A small number had siblings with autism (7 children). A specific question of ASD was the most common reason for referral (58 children [55%]; >1 option could be selected), followed by communication concerns (41 children [39%]), and behavioral and emotional concerns (24 children [23%]). Seventy-two children (68%) received a diagnosis of ASD by the MDT.

**Table 1.** Child Sample Demographic Information

Characteristic	Children, No. (%)		
	Total (N = 106)	With ASD diagnosis (n = 72 [68%])	Without ASD diagnosis (n = 34 [32%])
Age, mean (SD), mo	41.9 (13.3)	39.3 (12)	47.3 (14.5)
Gender			
Boys	79 (75)	57 (79)	22 (65)
Girls	27 (25)	15 (21)	12 (35)
Race and ethnicity			
Black or African	6 (6)	6 (8)	0
Chinese	7 (7)	5 (7)	2 (6)
Filipino	3 (3)	2 (3)	1 (3)
Latin American or Hispanic	4 (4)	3 (4)	1 (3)
Middle Eastern, Arab, or North African	5 (5)	3 (4)	2 (6)
Multiracial	18 (17)	15 (21)	3 (9)
South Asian	14 (13)	14 (19)	0
Southeast Asian	3 (3)	2 (3)	1 (3)
White or European	45 (42)	22 (31)	23 (68)
Missing	1 (1)	0	1 (3)
Interpreter used			
Yes	6 (6)	4 (6)	2 (6)
No	100 (94)	68 (94)	32 (94)
Reason(s) for referral			
Query ASD^a^	58 (55)	46 (64)	12 (35)
Communication concerns	41 (39)	31 (43)	10 (29)
Behavioral or emotional concerns	24 (23)	9 (13)	15 (44)
Developmental or cognitive concerns	23 (22)	16 (22)	7 (21)
Query ADHD^b^	13 (12)	5 (7)	8 (24)
Social concerns	12 (11)	8 (11)	4 (12)
Anxiety or obsessive compulsive disorder concerns	3 (3)	2 (3)	1 (3)
Eating concerns	2 (2)	1 (1)	1 (3)
Motor concerns	2 (2)	2 (3)	0
Missing	2 (2)	2 (3)	0
Sibling with autism			
Yes	6 (6)	5 (7)	1 (3)
No	100 (94)	67 (93)	33 (97)
Autism Diagnostic Interview, Revised score, mean (SD)			
No.	74	45	29
Social interaction score	11.4 (6.7)	14.2 (6.1)	7.1 (5)
Communication score	9.8 (4.5)	11.9 (3.9)	6.5 (3.4)
Restricted or repetitive behavior score	4.3 (2.8)	5.3 (2.6)	2.9 (2.3)
Autism Diagnostic Observation Schedule, 2nd edition			
Toddler	28 (26)	22 (31)	6 (18)
Module 1	32 (30)	29 (40)	3 (9)
Module 2	31 (29)	19 (26)	12 (35)
Module 3	15 (14)	2 (3)	13 (38)
Calibrated Severity Score, mean (SD)	6.9 (2.7)	8.1 (2)	4.3 (2.3)
Mullen Scales of Early Learning T-scores, mean (SD)			
Visual reception (n = 106)	36.1 (15)	30.5 (13.5)	47.4 (10.9)
Expressive language (n = 106)	32 (12.2)	27.7 (10.3)	41.3 (10.8)
Fine motor (n = 89)	32.9 (12.5)	29 (10.3)	41.1 (12.7)
Receptive language (n = 60)	31.9 (13.9)	25.7 (11.6)	41.7 (11.8)
Early learning composite (n = 53)	73.9 (21.5)	65 (19)	88.8 (16.9)

Abbreviations: ADHD, attention-deficit/hyperactivity disorder; ASD, autism spectrum disorder.

^a^ Query ASD included specific features within the diagnostic criteria (decreased eye contact, sensory issues, and restricted or repetitive behaviors).

^b^ Query ADHD included attention and hyperactivity concerns.

### General Pediatrician Sample

Demographic information for general pediatrician participants is provided in Table 2. Seventeen pediatricians (12 women [71%]) participated in the study with more than half (9 pediatricians [53%]) practicing for more than 10 years. Most identified no additional ASD training (11 pediatricians [65%]). Only 1 pediatrician never gave ASD diagnoses; most gave 1 or 2 diagnoses per month (13 pediatricians [76%]). Use of tools varied considerably among participants.

**Table 2.** General Pediatrician Sample Demographic Information

Characteristic	Pediatricians, No. (%) (N = 17)
Gender	
Woman	12 (71)
Man	5 (29)
Years in practice	
0-9	8 (47)
10-19	4 (24)
20-29	3 (18)
30-39	2 (12)
Extra training in child development^a^	
No	11 (65)
Yes	6 (35)
Mean wait time from referral to first appointment, wk	
1-4	8 (47)
5-8	3 (18)
9-12	1 (6)
>12	4 (24)
Missing	1 (6)
Frequency of suspected ASD cases, No./mo	
0-3	9 (53)
4-7	2 (12)
8-11	5 (29)
≥12	1 (6)
Frequency of ASD diagnoses given, No./mo	
None	1 (6)
1-2	13 (76)
3-5	1 (6)
≥6	2 (12)
Tool(s) typically used in practice	
Developmental questionnaires	10 (59)
Modified Checklist for Autism in Toddlers	8 (47)
ADOS-2 or mini-ADOS-2	4 (24)
Rapid Interactive Test for Autism–Toddlers	3 (18)
Childhood Autism Rating Scale	3 (18)
School questionnaires	3 (18)
Other questionnaires	1 (6)

Abbreviations: ADOS-2, Autism Diagnostic Observation Schedule 2nd Edition; ASD, autism spectrum disorder.

^a^ Additional training (could select more than 1) included workshops (n = 3), ADOS-2 or mini-ADOS-2 training (n = 2), Rapid Interactive Test for Autism–Toddlers training (n = 2), informal (n = 2), and additional rotations in developmental pediatrics in community pediatrics fellowship (n = 1). Mini-ADOS-2 refers to an abbreviated form of the ADOS-2 used among local pediatricians.

### Accuracy

Table 3 shows the agreement between the pediatrician and the MDT diagnosis. Overall percentage agreement was 0.76 (95% CI, 0.67-0.83). The κ value was 0.50 (95% CI, 0.34-0.67), indicating moderate agreement. Sensitivity was 0.75 (95% CI, 0.67-0.83), and specificity was 0.79 (95% CI, 0.62-0.91). The PPV and NPV were calculated for the observed prevalence of 67.9%, with a PPV of 0.89 (95% CI, 0.80-0.94) (ie, 89% agreement with the MDT) and NPV of 0.60 (95% CI, 0.49-0.70) (ie, 60% agreement with the MDT).

**Table 3.** Accuracy Results, Certainty, and Out-of-Study Pediatrician Actions

Variable	Cases, No. (%)			
	MDT diagnosis of ASD		MDT diagnosis not ASD	
	Pediatrician diagnosis correct (n = 54)	Pediatrician diagnosis incorrect (n = 18)	Pediatrician diagnosis correct (n = 27)	Pediatrician diagnosis incorrect (n = 7)
Pediatrician certainty, Likert scale				
1	0	0	0	0
2	1 (2)	3 (17)	1 (4)	2 (29)
3	10 (19)	7 (39)	11 (41)	3 (43)
4	19 (35)	7 (39)	13 (48)	1 (14)
5	24 (44)	1 (6)	2 (7)	1 (14)
MDT certainty, Likert scale				
1	0	0	0	0
2	0	0	3 (11)	0
3	2 (4)	3 (17)	9 (33)	3 (43)
4	11 (20)	7 (39)	14 (52)	2 (29)
5	41 (76)	8 (44)	1 (4)	2 (29)
Pediatrician action				
Tell family child has ASD	37 (69)	0	0	1 (14)
Tell family child does not have ASD	0	8 (44)	12 (44)	0
Refer to subspecialist	14 (26)	7 (39)	3 (11)	5 (71)
Watch and wait	3 (6)	2 (11)	4 (15)	0
Tell family child has different diagnosis	0	1 (6)	8 (30)	1 (14)

Abbreviations: ASD, autism spectrum disorder; MDT, multidisciplinary team.

We compared degrees of certainty between different agreement configurations (Table 3), assuming that the MDT represents the correct condition. Among true-positive cases (MDT and pediatrician agree the child has ASD), the pediatrician was certain or very certain 80% of the time (43 cases) and the MDT was certain or very certain 96% of the time (52 cases). As such, if pediatricians conferred ASD diagnoses when feeling certain or very certain, they would make 46 correct diagnoses and 2 incorrect diagnoses. Among true-negative cases (agreement the child does not have ASD), the pediatrician and MDT were both certain or very certain in 56% of cases (15 cases). In false-positive cases (pediatrician thought the child had ASD but the MDT did not), the pediatrician felt certain or very certain in only 29% of cases (2 cases) compared with 57% for the MDT (4 cases). Finally, for false-negative cases (MDT thought the child had ASD but the pediatrician did not), pediatricians were certain or very certain in 44% of cases (8 cases) compared with 83% for the MDT (15 cases). The MDT felt somewhat certain or not certain in 20 cases (19%).

Pediatricians indicated what they would have done had the child not been in this study (Table 3). Notably, in 69% of the true-positive cases (37 cases), pediatricians would have provided the child with an ASD diagnosis. In 44% of true-negative cases (12 cases), they would have told the family the child did not have autism; in 8 cases (30%), they would give alternative diagnoses (most commonly ADHD and language delay). The pediatrician would have given the child an ASD diagnosis in only 1 of the 7 false-positive cases and would refer to a subspecialist the majority of the time (5 cases [71%]). In false-negative cases, the pediatrician would incorrectly tell the family the child does not have autism 44% of the time (8 cases).

### Factors Associated With Accurate Assessment

Results of the logistic regression model estimating agreement within the sample diagnosed with ASD by the MDT are shown in Table 4. Pediatrician certainty was positively associated with accuracy (OR, 3.33; 95% CI, 1.71-7.34; *P* = .001) (Figure). Higher MSEL Visual Reception score was associated with worse accuracy (OR, 0.93; 95% CI, 0.89-0.97; *P* = .001), as was whether the child was speaking vs nonspeaking (OR, 0.17; 95% CI, 0.03-0.67; *P* = .03). ADOS-2 calibrated severity score was not a factor significantly associated with accuracy. Among the child’s demographic characteristics, gender and age were not factors significantly associated with accuracy of diagnosis. All children with a sibling with autism received an accurate diagnosis. White children had comparatively lower accuracy compared with children from minoritized racial and ethnic groups (OR, 0.32; 95% CI, 0.10-0.97; *P* = .04). We conducted post hoc analyses of the presenting features of 22 White vs 50 minoritized children in this sample. White and minoritized children in the sample were of comparable ages (mean, 40.7 vs 38.7 months), had similar ADOS-2 calibrated severity scores (mean, 7.5 vs 8.4), and similar MSEL Visual Reception scores (mean, 32.9 vs 29.6).

**Table 4.** Factors Associated With Accurate General Pediatrician Assessment for Children With an ASD Diagnosis

Variable	OR (95% CI)	*P* value	Sensitivity (95% CI)^a^
Pediatrician certainty, Likert score			
2	3.33 (1.71-7.34)	.001	0.26 (0.09-0.56)
5			0.93 (0.80-0.98)
Male gender	1.12 (0.28-3.88)	.90	NA
Sibling with ASD^b^			
No sibling with ASD	NA	NA	0.64 (0.52-0.74)
Sibling with ASD			1.00 (0.48-1.00)
Speaking ability			
Speaking	0.17 (0.03-0.67)	0.03	0.66 (0.51-0.78)
Nonspeaking			0.92 (0.73-0.98)
White or European race or ethnicity			
White or European	0.32 (0.10-0.97)	.04	0.59 (0.38-0.77)
Minoritized racial or ethnic group			0.82 (0.69-0.90)
Age	0.98 (0.93-1.02)	.30	NA
Autism Diagnostic Observation Schedule composite	1.15 (0.87-1.50)	.30	NA
Mullen Visual Reception t score^c^			
30	0.93 (0.89-0.97)	.001	0.81 (0.69-0.89)
50			0.49 (0.28-0.70)

Abbreviations: ASD, autism spectrum disorder; NA, not applicable; OR, odds ratio.

^a^ Logistic regression results are presented. Sensitivity refers to agreement within the sample diagnosed with ASD by the multidisciplinary team.

^b^ Logistic regression estimates could not be obtained because none of the participants with a sibling with ASD (n = 5) had an incorrect diagnosis. Exact binomial confidence interval estimates are shown.

^c^ Sensitivity is for a hypothetical sample of individuals with a Mullen t-score at the given value.

**Figure.**
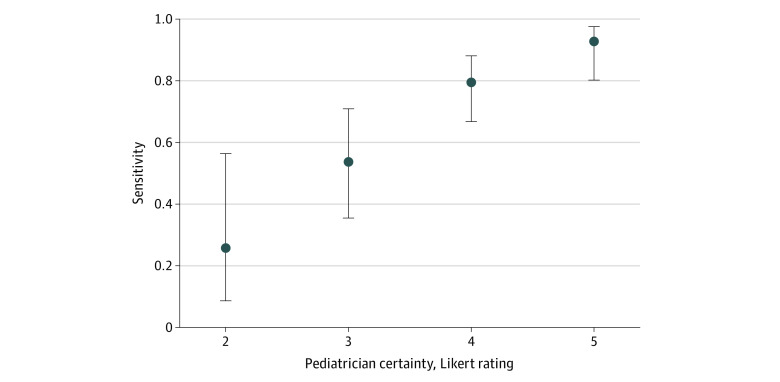
Sensitivity of General Pediatrician Autism Diagnosis by Pediatrician Certainty Sensitivity is on the vertical axis, and general pediatrician certainty Likert ratings from 1 to 5 are presented on the horizontal axis. There were no certainty ratings of 1. Means are presented with dots and 95% CIs with bars.

The logistic regression for specificity (agreement within the sample not diagnosed with ASD by the MDT) was on a comparatively smaller sample (34 cases) (eTable in Supplement 1). The only significant factor was having a sibling with autism.

## Discussion

This diagnostic study provides critical insights into the role that general pediatricians can play in ASD assessment. We used a prospective design with independent, contemporaneous assessments and randomization of assessment order. We assigned the MDT diagnosis as the correct condition; however, autism experts are not assumed to agree in all cases. Indeed, the MDT in our study felt somewhat certain or not certain in nearly 20% of cases. As such, perfect agreement is not a standard against which to judge our results.

Our results show that general pediatricians have a very high likelihood of correctly diagnosing ASD in children, particularly when they feel certain. When considering what pediatricians would have done outside the study, there was only 1 false-positive case where they would have assigned an ASD diagnosis. These results are comparable to those of a feasibility study^15^ of a general pediatrician diagnostic clinic indicating 93% agreement with expert psychologist assessment in a small sample of 14 children where the pediatrician indicated an ASD diagnosis was very likely. This contrasts with a study from New York,^25^ in which 87 children with an autism diagnosis from a community clinician (psychologist or pediatrician) underwent thorough assessment, and 20 of those children were found to not have autism. Importantly, many of the children without autism in that study^25^ had previously received a diagnosis of pervasive developmental disorder–not otherwise specified; as such, those results are difficult to interpret under the current *Diagnostic and Statistical Manual of Mental Disorders* (Fifth Edition) categorization of ASD. Our prospective design has added considerably to this evidence base, showing high agreement for cases identified with ASD by pediatricians.

NPV was considerably lower in our study. Equally concerning is that in 44% of false-negative cases, the pediatrician would have told the family the child did not have autism. One study^26^ has examined the ability of experts to detect features of ASD in a 10-minute video of a child. That study found that children with autism demonstrated atypical behavior only 11% of the time, and expert raters missed 39% of cases in the ASD group.^26^ As such, more caution is needed for pediatricians when definitively ruling out ASD, which might result in diagnostic delays.

In children with a diagnosis of ASD, we identified multiple factors associated with accurate classification by pediatricians. Co-occurring developmental delays (children who were nonspeaking and had lower Visual Reception scores) were associated with higher odds of accurate pediatrician assessment. Pediatrician certainty was a significant factor, whereas observable ASD features, measured by the ADOS-2, were not. Further exploration of factors associated with certainty are necessary. A possible correlate is the concept of frank autism, as described by de Marchena et al^27^ in their mixed-methods analysis of responses from 151 clinicians. Clinicians were more likely to see these cases in younger, less verbal children. Common signifiers in that study^27^ included motor mannerisms and atypical gait or posture, repetitive language, minimal social overtures, decreased eye contact, and unusual quality of speech. Importantly, the authors^27^ note that in such cases, more efficient diagnostic processes may be warranted.

We examined whether demographic factors were associated with accurate diagnosis of autism in children. Age and gender were not significant factors; however, our study focused on young children. Girls are known to receive a diagnosis of ASD later than boys,^28^ and our study would not have captured those identified in school age or adolescence. More work is needed to understand the diagnostic journey for gender-nonconforming children and youth. Surprisingly, our study found that children from minoritized racial and ethnic groups were more likely than White children to receive an accurate pediatrician assessment. In post hoc analyses, we examined whether these children had more extensive symptoms on presentation, indicating a higher threshold for identification and referral among minoritized children. Although minoritized children in our population tended to be younger and had higher ADOS-2 scores and lower MSEL scores than White children, none of these differences reached statistical significance. One aspect of assessment that could be explored in future studies is the impact of racial or ethnic concordance between patient and physician on diagnostic agreement.

### Limitations

This study has limitations that should be addressed. Our sample of pediatricians is not representative of all general pediatricians; this group was self-selected and, thus, was more likely to have an interest in ASD. However, they are likely representative of those who are more willing to provide ASD assessments. Owing to the small sample of pediatricians, we did not perform analyses of pediatrician-level factors associated with accuracy, which deserves future study. Several pediatricians saw only 1 child participant; as such, the random effect could not be estimated. To provide the same assessment battery to all participants, we focused only on young children. Similar study designs should be used to examine diagnostic accuracy in older children or youth. We were not able to complete the full MSEL with all children and prioritized the Visual Reception subscale as a proxy for nonverbal intelligence quotient, thus limiting access to other potentially important variables. There was a high proportion of children with ASD in our sample, which provided us with lower power to identify accuracy-related factors in children without autism. In addition, many general pediatricians in Canada operate in a consultant, rather than primary care role.^16^ Further analysis of practice type on diagnostic agreement may be warranted to support generalizability of results.

## Conclusions

In this diagnostic study, we found a high PPV for children assessed as having ASD by pediatricians, indicating that pediatricians should diagnose ASD when they feel certain. Higher pediatrician certainty and co-occurring developmental delays were associated with accurate assessment. Agreement was lower for cases where pediatricians did not think the child had autism; caution is needed before ruling out ASD.

## Notes

Supplement 1. **eTable.** Logistic Regression for Specificity (Agreement Within the Sample Not Diagnosed With ASD by the MDT)

Supplement 2.
Data Sharing Statement
