# Supplementary material for: Concordance of Diagnosis of Autism Spectrum Disorder Made by Pediatricians vs a Multidisciplinary Specialist Team
Source: JAMA Netw Open. 2023 Jan 25;6(1):e2252879. doi: 10.1001/jamanetworkopen.2022.52879 (PMC10187485; doi:10.1001/jamanetworkopen.2022.52879)
Supplement: Supplement 1. — eTable. Logistic Regression for Specificity (Agreement Within the Sample Not Diagnosed With ASD by the MDT) [file jamanetwopen-e2252879-s001.pdf]

Supplemental Online Content

Penner M, Senman L, Adoni L, et al. Concordance of diagnosis of autism spectrum disorder made by pediatricians vs a multidisciplinary specialist team. *JAMA Netw Open*. 2023;6(1):e2252879. doi:10.1001/jamanetworkopen.2022.52879

eTable. Logistic Regression for Specificity (Agreement Within the Sample Not Diagnosed With ASD by the MDT)

This supplemental material has been provided by the authors to give readers additional information about their work.

© 2023 Penner M et al. *JAMA Network Open.*

eTable. Logistic Regression for Specificity (Agreement Within the Sample Not Diagnosed With ASD by the MDT)

	**OR (95% CI)**	**p**	**Specificity (95%CI)**	
Pediatrician Certainty	2.21 (0.73,8.12)	.2		
Male	0.68 (0.09,3.84)	.7		
ASD Sib	-	-	*No ASD Sib* 0.78 (0.60,0.91)	*ASD Sib* 1.00 (0.16,1.00)
Verbal	2.08 (0.09,25.6)	.5		
Age	1.01 (0.95,1.07)	.7		
ADOS composite	0.98 (0.68,1.44)	.9		
Mullen VR t-score	1.01 (0.93,1.09)	.8		

© 2023 Penner M et al. *JAMA Network Open.*
