# Supplementary material for: Concordance of Diagnosis of Autism Spectrum Disorder Made by Pediatricians vs a Multidisciplinary Specialist Team
Source: JAMA Netw Open. 2023 Jan 25;6(1):e2252879. doi: 10.1001/jamanetworkopen.2022.52879 (PMC10187485; doi:10.1001/jamanetworkopen.2022.52879)
Supplement: Supplement 2. — Data Sharing Statement [file jamanetwopen-e2252879-s002.pdf]

Data Sharing Statement

Penner. Concordance of Diagnosis of Autism Spectrum Disorder Made by Pediatricians vs a Multidisciplinary Specialist Team. *JAMA Netw Open*. Published January 25, 2023. doi:10.1001/jamanetworkopen.2022.52879

Data
Data available: No

Additional Information
Explanation for why data not available: Data sharing requests will be considered upon reasonable request to the corresponding author.
